# Determination of the minimum fully protective dose of adenovirus-based DIVA vaccine against peste des petits ruminants virus challenge in East African goats

**DOI:** 10.1186/s13567-016-0306-4

**Published:** 2016-01-21

**Authors:** Barbara Holzer, Geraldine Taylor, Paulina Rajko-Nenow, Sophia Hodgson, Edward Okoth, Rebecca Herbert, Philip Toye, Michael D. Baron

**Affiliations:** The Pirbright Institute, Ash Road, Pirbright, Surrey, GU24 0NF UK; International Livestock Research Institute, Nairobi, Kenya

## Abstract

Peste des petits ruminants virus (PPRV) causes an economically important disease of sheep and goats, primarily in developing countries. It is becoming the object of intensive international control efforts. Current vaccines do not allow vaccinated and infected animals to be distinguished (no DIVA capability). We have previously shown that recombinant, replication-defective, adenovirus expressing the PPRV H glycoprotein (AdH) gives full protection against wild type PPRV challenge. We have now tested lower doses of the vaccine, as well as AdH in combination with a similar construct expressing the PPRV F glycoprotein (AdF). We show here that, in a local breed of goat in a country where PPR disease is common (Kenya), as little as 10^7^ pfu of AdH gives significant protection against PPRV challenge, while a vaccine consisting of 10^8^ pfu of each of AdH and AdF gives apparently sterile protection. These findings underline the utility of these constructs as DIVA vaccines for use in PPR control.

## Introduction

Peste des petits ruminants (PPR) is an important disease of sheep and goats which has recently become a major international target for improved control, marked by the adoption in 2014 of a resolution by the World Organisation for Animal Health (OIE) to establish a control programme with a view to eventual eradication of the disease [1]. The disease is caused by a morbillivirus, PPR virus (PPRV), closely related to the human pathogen measles virus (MV), as well as other animal pathogens such as canine distemper virus (CDV) and rinderpest virus (RPV). PPRV is widely distributed through large parts of Africa, the Middle East and Asia and is responsible for significant economic losses, primarily in developing countries [2–5].

Disease control is mostly achieved through the use of clinical or laboratory-based diagnosis coupled with vaccination. All the vaccines currently in use are attenuated strains of PPRV [6, 7]; these vaccines are effective, though they do not provide a DIVA (distinguishing infected from vaccinated animals) capability. These vaccines cause what is essentially a subclinical infection with PPRV, and therefore the antibody signatures of vaccinated and previously-infected animals are identical. Several alternative DIVA vaccines have been proposed based on recombinant viruses [8–13]. We have shown that recombinant replication-defective human adenovirus type 5 (Ad5) expressing the H surface glycoprotein of PPRV can act as a DIVA vaccine, inducing good levels of antibodies and protecting goats from experimental challenge with a pathogenic PPRV 4 months post vaccination [9]. Similar constructs have also been shown to be immunogenic in other studies [10, 11] or, more recently, both immunogenic and protective in sheep [8]. We have carried out an extended study of such recombinant adenovirus constructs in goats in East Africa, an area where PPR is endemic. Using local animals, we have analysed the immunogenicity and protective efficacy of different doses of vaccine, in order to determine the minimum protective dose. We have also compared the protection induced by Ad5-H alone to that induced by a vaccine combining Ad5-H with a similar construct expressing the other PPRV surface glycoprotein, F.

## Materials and methods

### Cells and viruses

Vero cells expressing the canine version of the morbillivirus receptor SLAM (signalling lymphocyte activation molecule) (vero-dog-SLAM, VDS) were obtained from Dr Paul Duprex, then at Queen’s University Belfast, N. Ireland, and maintained in Dulbecco’s modified Eagle’s medium containing 25 mM HEPES buffer, penicillin (100 U/mL), and streptomycin (100 µg/mL) (DMEM) containing 10% foetal calf serum (FCS). Zeocin was included at 0.1 mg/mL to maintain selection for SLAM expression. PPRV Ivory Coast/89 isolate [14] and recombinant PPRV rPPRV-GFP [15] were propagated and titrated in VDS cells. Titres were determined as the 50% tissue culture infectious dose (TCID_50_), calculated by the method of Spearman and Kärber [16]. Recombinant adenoviruses expressing ovine IL-2 (AdIL-2), PPRV H (AdH) or PPRV F (AdF), as well as the control adenovirus construct expressing GFP (AdGFP) were those previously described [9].

### Animal study

The animal study was approved by the Institutional Animal Care and Use Committee (IACUC) and Institutional Biosafety Committee (IBC) at the International Livestock Research Institute, Nairobi (ILRI) and the National Biosafety Authority (NBA) in Kenya. Forty-eight locally acquired goats (Small East African Goat breed) were housed in a containment barn that was proofed against insect and tick vectors. All the animals were tested immediately prior to vaccination for the presence of anti-PPRV antibodies using the PPRV H protein-specific cELISA, and found to be negative. The animals were divided into eight groups of six animals, which were vaccinated as described in Table 1. Blood samples for the preparation of serum were taken before vaccination and at 2, 3, 4 and 12 weeks post vaccination. At 12 weeks post vaccination, all remaining animals (*n* = 42) were challenged with 2 × 10^5^ TCID_50_ of PPRV Ivory Coast/89 [9, 14] delivered intranasally. Rectal temperatures were recorded daily, and the animals monitored daily for clinical signs, for 14 days after challenge. Blood was taken in EDTA on days 0, 2, 4, 6, 8, 10, 12 and 14 days post challenge and stored at −70 °C until tested for the presence of PPRV RNA. Samples of blood were taken at 7 and 14 days post infection to prepare serum.

**Table 1:** **Amounts of each recombinant adenovirus given to each animal in the experimental groups**

Group number	Vaccine
1	10^7^ AdH + 10^7^ AdGFP
2	10^8^ AdH + 10^8^ AdGFP
3	10^7^ AdH + 10^7^ AdIL-2
4	10^8^ AdH + 10^8^ AdIL-2
5	10^7^ AdH + 10^7^ AdF
6	10^8^ AdH + 10^8^ AdF
7	10^7^ AdH + 10^7^ AdF + 10^7^ AdIL-2
8	10^8^ AdGFP + 10^8^ AdIL-2

Viruses were diluted in PBS and delivered intramuscularly in a volume of 1 mL per animal.

### Other assays

Assays for serum antibodies specific for the PPRV H glycoprotein and the PPRV nucleocapsid (N) protein, for PPRV neutralising antibodies were as previously described [9] Viral RNA was assayed in 6% of the RNA extracted from 100 μL EDTA blood, using reverse transcription-real time PCR as described [17].

### Statistical analyses

Comparisons between groups were performed using linear mixed models in which animals were taken to be random factors and the other factors as fixed; calculations were performed using the *nlme* package in R. Multiple comparisons were carried out by using the Tukey corrected 95% confidence intervals as implemented in the R package *multcomp*, and quoted probability (*p*) values are from that package.

## Results

Previous studies demonstrated that a single dose of 10^9^ pfu of AdH induced complete protection against PPRV, when goats were challenged 4 months after vaccination. Therefore, the present animal study was designed to determine if (i) the AdH vaccine is still protective at 10^8^ or 10^7^ pfu per animal; (ii) it delivers improved protection when combined with AdF (as suggested by [10]); (iii) there is an adjuvant effect of co-expressing IL-2, as suggested by some data from our previous study [9]. Seven groups, of six animals each, were vaccinated with different amounts and combinations of replication-defective adenovirus constructs as described in “Materials and methods” section, with an eighth group kept as control animals. Six animals of the total 48, from different groups, died of an unspecified respiratory infection between vaccination and challenge, so only partial serology data is available for those animals.

**Figure 1:**
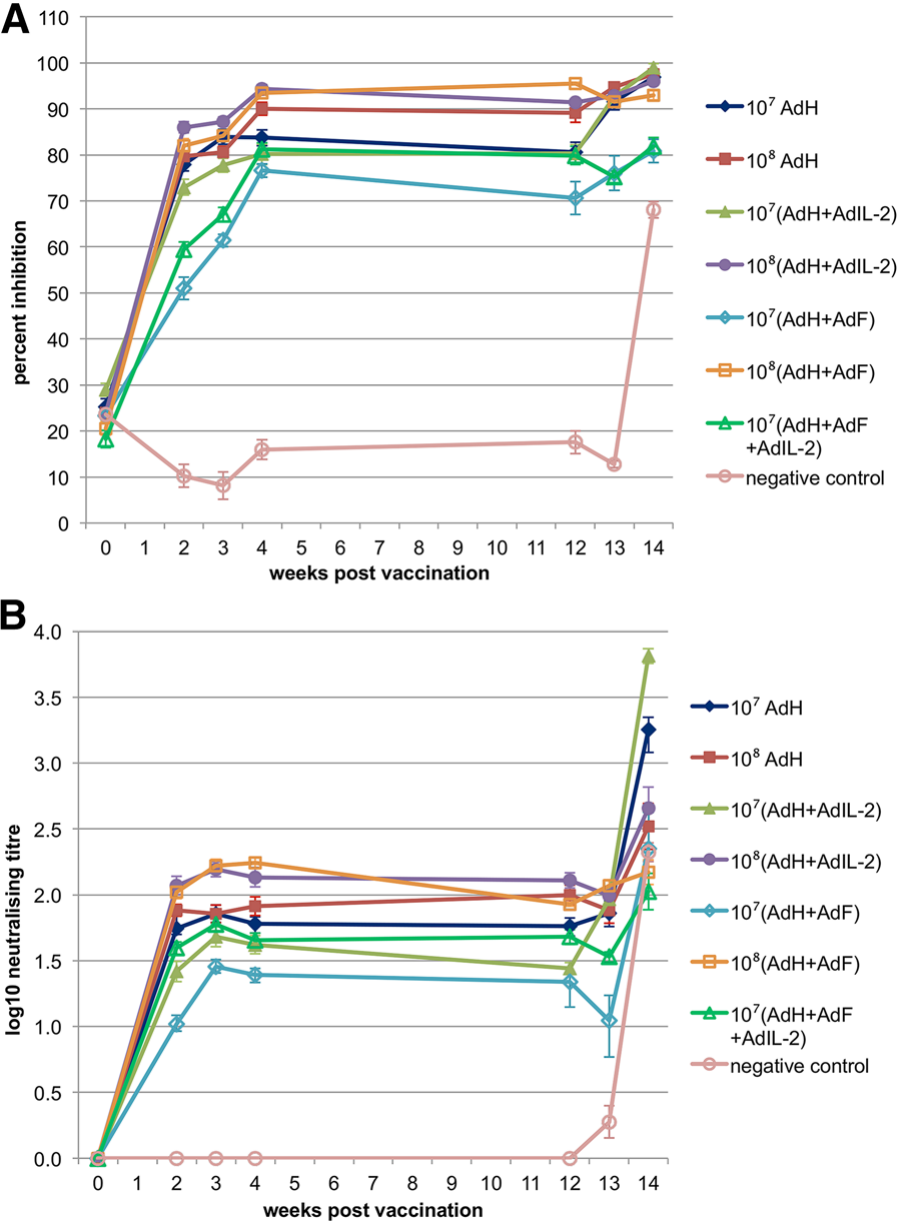
**Antibody responses to recombinant adenovirus vaccine.** The serum antibody response was determined after vaccination (blue bars) and after challenge (pink bars) as **A** the percent inhibition in the anti-H cELISA and **B** the PPRV neutralisation titre. Error bars are one standard error of the mean (SEM).

All the vaccinated animals developed a strong antibody response to the PPRV surface glycoprotein H, as measured by competition ELISA [18] (Figure 1A). This was true even of those animals given the lowest dose of vaccine (10^7^ pfu per animal). Groups 5 (10^7^ pfu each of AdF and AdH) and 7 (10^7^ pfu each of AdF, AdH and AdIL-2) showed significantly lower H antibody response to vaccination as measured in the cELISA, although still strongly positive (>70% inhibition). Group 5 showed a lower response than all groups except group 7 (*p* < 0.001 for comparison with groups 1, 2, 3, 4 and 6), while group 7 showed a lower response than groups 1, 2, 4 and 6 (*p* = 0.003 for comparison with group 1 and *p* < 0.001 for the rest). In the absence of AdF, there was no significant difference between the anti-H antibody response in animals given 10^7^ or 10^8^ pfu AdH (group 1 vs group 2, and group 3 vs group 4). The addition of AdIL-2 had no effect on induction of anti-H antibody (comparing groups 1 and 3 or groups 2 and 4), although it did improve the antibody response to low dose AdH + AdF (comparison of groups 5 and 7). As expected, antibodies to PPRV H protein were not detected in the negative control animals until the normal response to the challenge virus at 14 days post infection (dpi) (Figure 1A).

PPRV-specific serum neutralising antibodies were also analysed (Figure 1B). The two assays gave broadly comparable results, although the cELISA was more sensitive. Again, group 5 showed lower antibody responses during the period prior to challenge than the other groups, although this time there was no statistically significant difference between groups 5 and 3. Group 7 animals showed a significantly higher neutralising antibody response than group 5 (*p* = 0.034), but were not significantly different to any of the other groups. Group 3 was significantly lower than groups 4 and 6. Notably, inclusion of AdF in the vaccination inoculum either reduced the neutralising antibody response (group 1 vs group 5) or made no difference (group 2 vs group 6; group 3 vs group 7).

**Figure 2:**
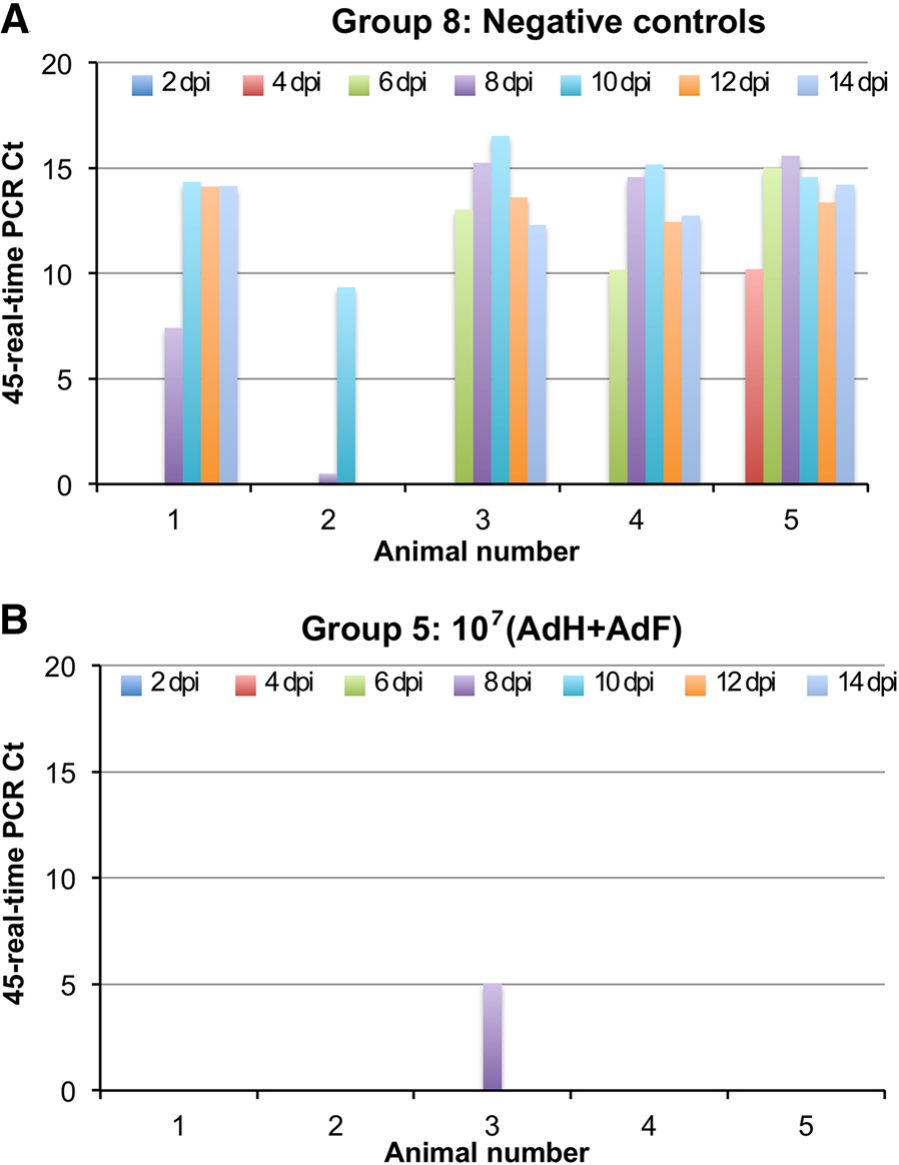
**PPRV viraemia after challenge.** Blood was collected from all animals at 2, 4, 6, 8, 10 and 12 dpi and stored at −70 °C until assay. Real-time PCR was used to measure PPRV RNA, which is plotted as 45-Ct, where Ct is the threshold cycle. **A** data from negative control animals; **B** data from group 5 animals. All other samples were completely negative for PPRV RNA.

All animals were challenged with 10^4^ TCID_50_ of wild type PPRV Ivory Coast/89 at 12 weeks after vaccination. This isolate caused severe disease in UK goats [14]. However, in the local breed of goats available in Nairobi, the clinical response was almost undetectable, with minimal temperature changes and no sustained clinical signs. In order to resolve the extent of protection provided by the vaccine, we therefore considered the presence or absence of PPRV RNA in blood samples during the challenge (taken as a measure of viraemia) and the immune response to other PPRV proteins, an extremely sensitive measure of virus replication, even in the absence of clinical signs of disease. All five unvaccinated control animals developed clear viraemia (Figure 2A). In contrast, none of the vaccinated animals showed any detectable viraemia, apart from one animal in group 5, on 1 day (Figure 2B). All other samples were negative.

**Figure 3:**
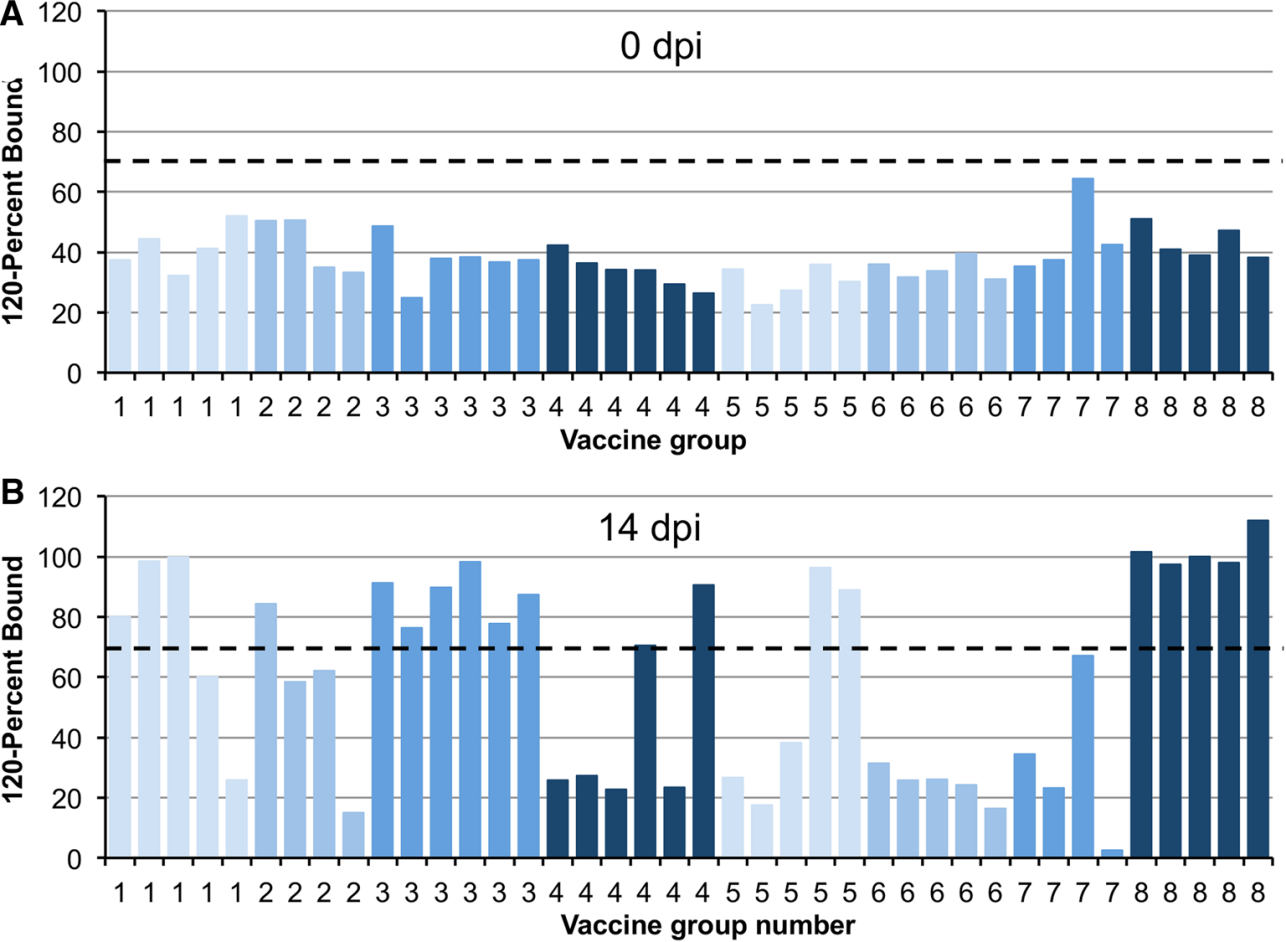
**Development of antibodies to PPRV N protein.** Serum samples before (**A**) and 14 days after (**B**) challenge were assayed for anti-N antibodies using the N protein specific cELISA. In this assay, the read-out is in percent of the monoclonal that remains bound (PB), so it has been plotted as 120-PB so that the plotted value increases as the amount of anti-N antibody increases. Values of (120-PB) >70 (values of PB <50) are considered positive (dashed line).

The development of antibodies to the PPRV N protein was determined using the N protein-specific competition ELISA (Figure 3). As expected, all the unvaccinated control animals developed a strong anti-N response by 14 dpi. In addition, varying numbers of animals in the other experimental groups seroconverted to the PPRV N protein, showing at least some replication of the virus (Table 2). Most of the animals in groups 1 and 3 (10^7^ AdH) were N antibody positive. The only groups in which no animals were observed to seroconvert in response to N protein were group 6 (10^8^ of both AdH and AdF) and group 7 (10^7^ of each of AdH, AdF and AdIL-2).

**Table 2:** **Development of antibodies to other PPRV proteins**

Group number	Number positive for anti-N antibodies/Number in group
1	3/5
2	1/4
3	6/6
4	2/6
5	2/5
6	0/5
7	0/4
8	5/5

Numbers of animals in each group that were positive ((120-PB) >70%) for antibodies against PPRV N protein.

## Discussion

Comparison of the immunogenicity and protective efficacy of different doses of Ad-expressed PPRV vaccines demonstrated that the recombinant adenovirus expressing the PPRV H, previously shown to be protective at a dose of 10^9^ pfu per animal [9], is also protective at 10^8^ pfu per animal (no detectable viraemia, limited replication of challenge virus). At 10^9^ pfu per animal, in addition to protection from clinical PPRV, we also found complete suppression of viraemia, although all the animals developed anti-N antibodies, showing that this test is an extremely sensitive test for replication of the challenge virus. At the lowest dose of AdH (10^7^ pfu per animal), there was still a strong antibody response, and no viraemia, but more (3 out of 5) of the animals developed anti-N antibodies.

Our data support the suggestion that the combination of AdH and AdF may be more protective than AdH alone, since group 6 restricted the challenge virus better than group 2, although the differences were marginal. However, we did not observe the increased PPRV-neutralising titre seen by Wang et al. [10] in goats vaccinated with an adenovirus expressing PPRV H and F. A significant difference between these two studies is that, in the present case, we expressed each protein separately, so each had the possibility to fold into its native conformation. In the study by Wang et al. [10], F and H were expressed as a single fusion protein. Under these circumstances, neither protein is likely to fold normally. It has been shown that such misfolded proteins can be bound directly by MHC class II [19]; in addition, such a protein will be removed from the ER by the normal quality control system (reviewed in [20]) and degraded by the ER-associated protein degradation system, rapidly generating F/H protein-derived peptides which can be presented by both class I and class II systems [21, 22]. Such processes may have led to the higher levels of anti-PPRV antibody observed in response to their (F + H) fusion protein compared to AdF or AdH alone.

While it may not have played a role in their reported increased antibody response to Ad(F + H) compared to AdH, Wang et al. also vaccinated goats with two doses of adenovirus-vectored vaccine, whereas we have considered it desirable to have a vaccine that functions after a single dose, since this will be more practical in developing countries where the major cost and effort is in delivering vaccine to the animals.

The overall conclusion was therefore that the vaccine consisting of 10^8^ (AdH + AdF) appears to be completely effective and provides a DIVA vaccine capability when used in conjunction with the existing commercial cELISAs, which recognise antibodies to PPRV H or PPRV N. The protection was complete at 3 months post vaccination and with only a single dose of vaccine. However, a longer term study (at least 1 year) would be advisable to establish the duration of protection and the duration of detectable marker antibody (anti-H antibody).

A very interesting observation was that there appears to be a significant difference in the pathogenicity of the PPRV isolate in UK and E. African goats. Although this isolate (Ivory Coast/89) has been clearly virulent in studies at TPI, the same virus had little effect on the native breed of goats used at ILRI; transient high temperatures were seen in several of the control animals, and none in the vaccinated animals (not shown), but this was not consistent across the group. Mild clinical signs were sometimes observed in different animals, but it was clear that the animals were not being kept isolated from other infectious agents, as six animals died during the time between vaccination and challenge, and the significance of slight nasal discharge for 1 day is limited. The reasons for the lack of severe clinical signs of disease in the control animals, despite clear virus replication, and prolonged viraemia in most cases, are not clear. One possibility is that there is significant and general resistance to PPRV in the breed of goat used or, alternatively, resistance may be due to an interaction of virus and host strain, so that viruses may appear pathogenic or mild depending on what hosts they have recently been in (the challenge strain had been passaged in UK goats). This has implications for surveillance for disease, as virus may be mild or inapparent when first entering a new country or an area with a different breed of goats or sheep.

Studies are necessary in local breeds of goats using local isolates of PPRV as well as other known pathogenic isolates. If some animals are able to support virus growth, and possibly spread, without showing significant clinical signs, this would make control measures based on clinical surveillance alone insufficient.
